# Straightforward synthesis of Sulfur/N,S-codoped carbon cathodes for Lithium-Sulfur batteries

**DOI:** 10.1038/s41598-020-61583-1

**Published:** 2020-03-17

**Authors:** Marta Sevilla, Jorge Carro-Rodríguez, Noel Díez, Antonio B. Fuertes

**Affiliations:** Instituto de Ciencia y Tecnología del Carbono, INCAR-CSIC. Francisco Pintado Fe 26, Oviedo, 33011 Spain

**Keywords:** Batteries, Batteries

## Abstract

An upgrade of the scalable fabrication of high-performance sulfur-carbon cathodes is essential for the widespread commercialization of this technology. Herein we present a simple, cost-effective and scalable approach for the fabrication of cathodes comprising sulfur and high-surface area, N,S-codoped carbons. The method involves the use of a sulfur salt, *i.e*. sodium thiosulfate, as activating agent, sulfur precursor and S-dopant, and polypyrrole as carbon precursor and N-dopant. In this way, the production of the porous host and the incorporation of sulfur are combined in the same procedure. The porous hosts thus produced have BET surface areas in excess of 2000 m^2^ g^−1^, a micro-mesoporous structure, as well as sulfur and nitrogen contents of 5–6 wt% and ~2 wt%, respectively. The elemental sulfur content in the composites can be precisely modulated in the range of 24 to *ca*. 90 wt% by controlling the amount of sodium thiosulfate used. Remarkably, these porous carbons are able to accommodate up to 80 wt% sulfur exclusively within their porosity. When analyzed in lithium-sulfur batteries, these sulfur-carbon composites show high specific capacities of 1100 mAh g^−1^ at a low C-rate of 0.1 C and above 500 mAh g^−1^ at a high rate of 2 C for sulfur contents in the range of 50–80 wt%. Remarkably, the composites with 51–65 wt% S can still provide above 400 mAh g^−1^ at an ultra-fast rate of 4 C (where a charge and discharge cycle takes only ten minutes). The good rate capability and sulfur utilization was additionally assessed for cathodes with a high sulfur content (65–74%) and a high sulfur loading (>5 mg cm^−2^). In addition, cathodes of 4 mg cm^−2^ successfully cycled for 260 cycles at 0.2 C showed only a low loss of 0.12%/cycle.

## Introduction

In the quest for high energy/high power density energy storage systems capable of powering electric vehicles, Lithium-Sulfur batteries have emerged as one of the most promising alternatives. This is due to their large theoretical gravimetric and volumetric energy densities (2600 Wh kg^−1^ and 2800 Wh L^−1^, respectively) compared to those provided by current Li-ion technology (240 Wh kg^−1^ and 670 Wh L^−1^), as well as their inherent lower cost (~$150/Ton S *vs*. ~$10000/Ton LiCoO_2_)^1,2^. Moreover, sulfur is the tenth most abundant element in the Earth’s crust which ensures its long-term supply, and it is relatively nontoxic and environmentally benign in contrast to Li-ion cathodes such as LiCoO_2_. However, the application of this technology faces a number of important challenges. Both S and the end-product Li_2_S are poor electronic/ionic conductors; a high-volume expansion/shrinking takes place during the lithiation and delithiation processes (*i.e*., 80%) and active material is continuously lost during cycling due to the dissolution and migration of the intermediate lithium polysulfides, LiPSs (Li_2_S_x_, 4 ≤ x ≤ 8) (commonly known as the “shuttle effect”)^1,3,4^. All these phenomena lead not only to an inefficient use of the sulfur that limits the practical specific capacity, but also to low coulombic efficiencies and premature failure of the batteries. To solve these problems, optimization of the sulfur cathode has been undertaken following a variety of strategies. One of the most widespread is the immobilization of sulfur in a carbon material due to the fact that this kind of material comprises several suitable characteristics: (i) a high electronic conductivity; (ii) a high chemical and electrochemical inertness; (iii) a porous structure capable of confining the S, buffering the changes in volume during sulfur lithiation and delithiation and confining the LiPSs formed; and (iv) an easy heteroatom doping (*e.g*. O, S, N) that facilitates the chemical fixation of the LiPSs. As part of this strategy, a portfolio of carbon scaffolds has been analyzed, including graphene, carbon nanotubes, carbon nanofibers and porous carbons with different pore characteristics (microporous carbons, mesoporous carbons, carbons with hierarchical porosity, *etc*.)^3,5^. In particular, porous carbons are by far the most studied sulfur hosts on the grounds of their easy-to-design pore structure, tunable surface chemistry and potential low-cost. Indeed, control of both their pore structure and their surface chemistry is key to achieving a high sulfur utilization and stable performance. In this regard, microporous carbons with a narrow pore size distribution in the range of 0.5–1.0 nm offer a strong interaction with LIPSs resulting in a very stable prolonged cycling^6–8^. However, the limited availability of pore volume restricts the maximum sulfur content to values below 50%, limiting the energy density of the device. In contrast, mesoporous carbon materials offer high pore volumes for high sulfur loading, a better volume change accommodation and enough space for Li^+^ migration, but fail to prevent the dissolution of polysulfides^5,9^. Therefore, hierarchical pore structures that combine the merits of both micro- and mesopores are preferable. On the other hand, heteroatom doping (N, S, P, O, *etc*.) increases the polarity of the carbon material and thereby enhances the adsorption of polar LiPSs and Li_2_S, improving long-term cycling stability^10,11^. Furthermore, recent studies have shown that N- and S-moieties do not have just an anchoring ability, but electrocatalytic activity thereby improving LiPS redox kinetics^12–14^.

The conventional methods employed for the fabrication of sulfur/carbon composites comprise firstly the synthesis of the porous carbon host. This can be achieved by means of a variety of techniques, from complicated ones involving multiple steps such as nanocasting techniques^5,15^, to relatively simpler ones, including traditional chemical or physical activation processes^16^. Afterwards, sulfur can be infiltrated *via* melt diffusion^17,18^, vapor phase infiltration^19^, solution infiltration^20^, mechanical intrusion (*e.g*., ball-milling)^21^ or wet chemical processes (using a soluble sulfur-containing compound, such as sodium thiosulfate, sodium sulfide, sodium polysulfide, *etc*.)^16,22–25^. Many of these techniques have certain drawbacks. For example, sulfur is only soluble in a few substances, which are also highly toxic, such as CS_2_, benzene or toluene. On the other hand, mechanical intrusion is only appropriate in the case of carbon hosts with large external surfaces (as sulfur cannot be introduced into narrow pores) and the linkage between sulfur and the carbon host is weak^26^. Melt infiltration is the most widely adopted methodology owing to its simplicity compared to the other approaches. However, this technique also has drawbacks, such as the fact that the small micropores are not used to full advantage while severe aggregation of sulfur can occur in the macropores, limiting sulfur utilization and the transfer of electrons^25^. Therefore, novel methodologies for the production of sulfur/carbon composites with the homogeneously distributed sulfur throughout the carbon host are highly desirable. From the scaling-up point of view, the simplification of the fabrication process by combining the production of the porous host and the incorporation of sulfur in the same procedure would be highly advantageous, since this would reduce the number of steps needed. Furthermore, if the same substance were used to produce pores and sulfur, the amount of waste would also be reduced. In this regard, Li *et al*. demonstrated the *in situ* synthesis of sulfur nanoparticles in 3D porous carbon by the thermal treatment of a mixture of glucose as carbon precursor, NaCl as template and Na_2_S as template and sulfur precursor^23^. The assembly of the structure was induced by freeze-drying, followed by a high-temperature treatment to produce a 3D structure in which the NaCl-Na_2_S self-stacked nanoparticles are enclosed by a film of glucose-derived carbon. Finally, immersion of the mixture in an aqueous solution of Fe(NO_3_)_3_ led to the dissolution of the NaCl template, resulting in the production of macropores and the *in situ* generation of sulfur nanoparticles. More recently, Luo *et al*. followed a similar approach, in which Na_2_SO_4_ was used as template and sulfur precursor, glucose as carbon precursor and a melamine foam as 3D template to avoid the aggregation of Na_2_SO_4_-glucose which impedes the formation of sulfur^25^. However, both strategies rely on the use of a soluble precursor to fabricate the porous structure by inducing the subsequent crystallization of the salt templates by means of freeze-drying. Besides, the pore development achieved is quite limited, so that most of the sulfur is located outside the porosity of the materials. Therefore, further research along these lines is still necessary. In this regard, we have recently developed a methodology for the fabrication of sulfur-carbon composites based on the use of sodium thiosulfate as both activating agent and sulfur precursor^27^. In these composites, the carbon host is highly porous (S_BET_ > 1900 m^2^ g^−1^) and the elemental sulfur is generated *in situ* inside the porosity of the carbon particles. However, as in previous works, a soluble carbon precursor, *i.e*. tannic acid, and a solution/freeze-drying process were used. In the present research work we investigate the general applicability of this strategy to non-soluble precursors by means of a simple and scalable solid-phase mixing of the different ingredients. As carbon precursor, we rationally selected a N-rich polymer such as polypyrrole in order to fabricate a N-doped host with polysulfides trapping ability and a micro-mesoporous structure, since we have previously shown that N-rich substances can lead to micro-mesoporous materials in chemical activation approaches^28–30^. Moreover, we show that sodium thiosulfate has a third function as S-dopant, yielding sulfur/N,S-codoped carbon composites in which a sulfur fraction of up to 80% can be embedded inside the porosity of the carbon. As a result, these materials offer a good performance as cathodes in Li-S batteries, with high specific capacities of 1100 mAh g^−1^ at a low rate and as much as 500 mAh g^−1^ at a high rate of 2 C for S contents in the composite of up to 83 wt%. Cathodes comprising both a high sulfur content (65–74%) and a high sulfur loading (5.3–5.7 mg cm^−2^) also show a good rate capability and sulfur utilization, with specific capacities of 930–1000 mA h g^−1^ S (5.3 mA h cm^−2^) at 0.05 C and as much as 300–400 mAh g^−1^ S at a rate of 1 C. Their long-term stability is demonstrated by a capacity retention of 70% after 260 cycles at 0.2 C (a loss of 0.12%/cycle).

## Results and Discussion

### Structural, textural and chemical properties of the porous carbon host and the sulfur/carbon composites

**Figure 1:**
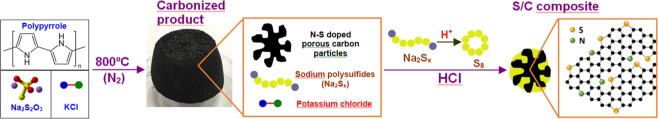
Schematic illustration of the synthesis procedure for the fabrication of the sulfur-carbon composites.

The synthesis procedure is illustrated in Fig. 1. In this synthesis strategy, sodium thiosulfate is used as both activating agent and sulfur precursor, which makes it possible to reduce the number of steps involved in the synthesis procedure, and decrease the amount of chemical products consumed and quantity of waste produced. Importantly, an inert and benign salt such as KCl is used as confinement medium to favor the activation reactions and enhance the development of porosity on the carbon material. This enhancement of porosity allows it to accommodate a greater amount of sulfur (*vide infra*). The rational selection of a N-rich polymer, in this case polypyrrole, allows the N-doping of the carbon host, while S-doping is taking place during the activation process, the result of which is a composite consisting of sulfur embedded in a N,S-codoped highly porous carbon material with a micro-mesoporous structure (*vide infra*).

As we have recently shown, when a mixture of a carbon precursor, sodium thiosulfate and potassium chloride is heat-treated under an inert atmosphere up to 800 °C, several consecutive processes take place: (1) decomposition of sodium thiosulfate into sodium sulfate (at *ca*. 300 °C), (2) pyrolysis of the carbon precursor, (3) redox reaction between the pyrolyzed precursor and sodium sulfate (>500 °C) which generates porosity within the carbon and (4) melting of the potassium chloride (770 °C) to provide a confined reaction medium^31^. The carbonization product is a mixture of porous carbon and a solidified product composed of KCl and sodium sulfide/polysulfides obtained as activation by-products. When this solid product is immersed in water, all the inorganic impurities (KCl and Na_2_S_x_) are dissolved leaving porous carbon particles, which can easily be collected by filtration. However, if the solid product is immersed in an aqueous acidic solution (*e.g*., HCl), alongside the dissolution of the inorganic species (KCl and Na_2_S/Na_2_S_x_), the disproportionation of polysulfide anions takes place, generating elemental sulfur and hydrogen sulfide:

1 $${{\rm{Na}}}_{2}{{\rm{S}}}_{{\rm{x}}}+2\,{\rm{HCl}}\to ({\rm{x}}-1)\,{\rm{S}}+{{\rm{H}}}_{2}{\rm{S}}+2\,{\rm{NaCl}}$$

**Figure 2:**
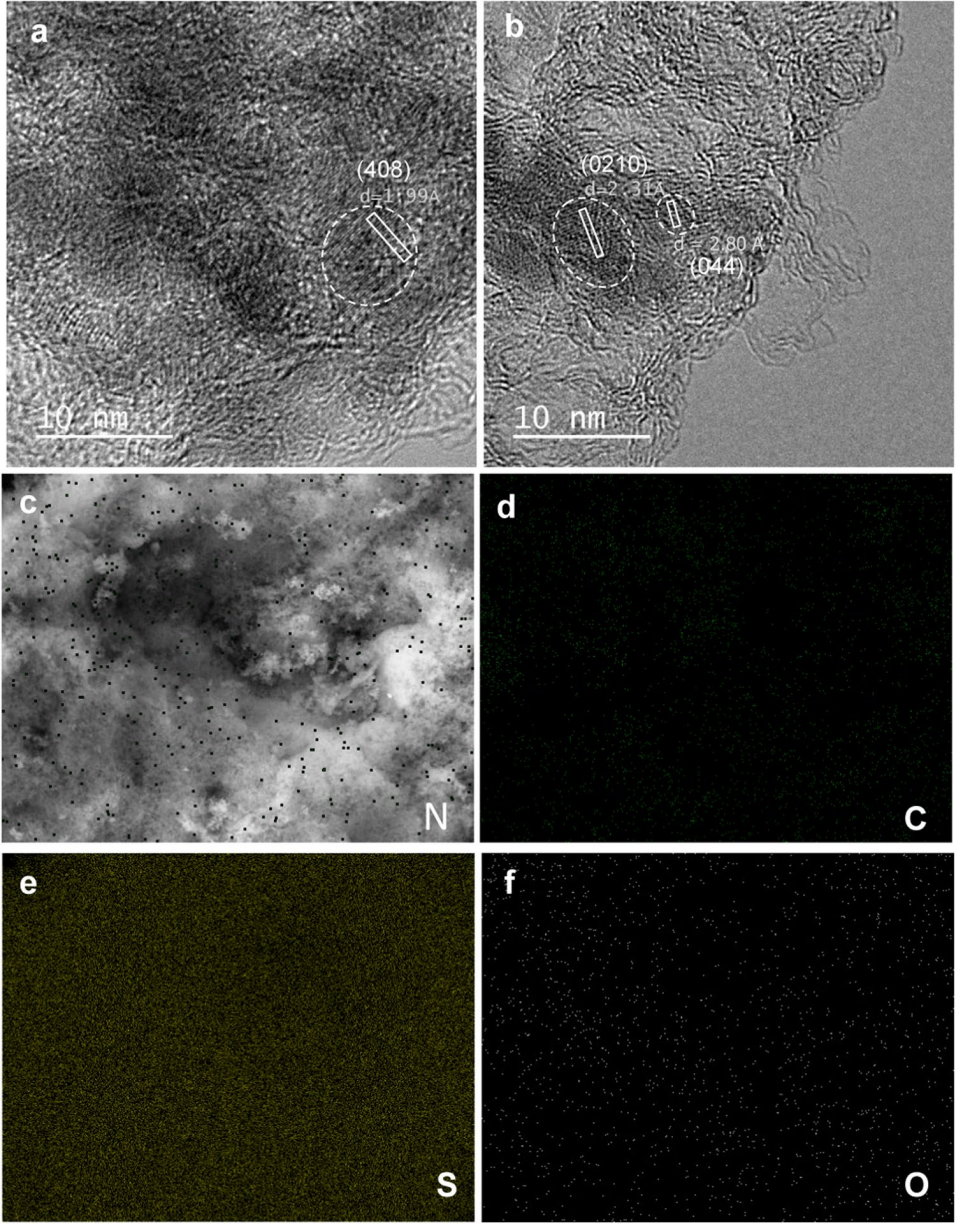
(**a**,**b**) HRTEM images of the sample CPS74, and SEM-EDX mappings of (**c**) nitrogen, (**d**) carbon, (**e**) sulfur and (**f**) oxygen collected from the area shown in (**c**) for the sample CPS51.

Since the dissolved polysulfides diffuse inside the pore network as a consequence of capillary forces, the *in-situ* formed sulfur nanoparticles are uniformly accommodated inside the porosity of the carbon material^27^. This is supported by the results of the HRTEM analysis of the S/C composite containing 74% S (*i.e*. CPS74) presented in Fig. 2a,b. In these images, the lattice fringes corresponding to interplanar spacings of 1.99, 2.31 and 2.80 Å, ascribed to the (408), (0210) and (044) planes of orthorhombic sulfur, are clearly visible. The SEM-EDX mapping in Fig. 2e shows the uniform distribution of S throughout the particles. Moreover, as an N-rich precursor has been used (16.5 wt% N), the resulting S/C composite is doped with a certain amount of nitrogen heteroatoms, as evidenced by the N mapping of Fig. 2c. This is further confirmed by the SEM-EDX mapping (Fig. S1a) and elemental analysis (2 wt% N, Table 1) of the corresponding carbon host. Also worth noting is that both the EDX and elemental analysis reveal an additional S-doping of the carbon host, *i.e*. 5.6 wt% (Table 1 and Fig. S1c), which is a consequence of secondary reactions with sodium sulfate^32^. The presence of N and S heteroatoms may favor the retention of polysulfides owing to the enhanced interaction between them and the polar polysulfides. Moreover, it has been demonstrated that dual doping with N and S heteroatoms has a synergistic effect due to the redistribution of the electron density within the carbon host that strengthens the interactions between the carbon and polysulfides^33^. Further analysis of the nature of the N- and S-groups in the carbon material was performed by XPS. Figure S2 shows the corresponding N 1 s and S 2p high resolution core level spectra corresponding to the CP51 sample. Deconvolution of the N 1 s spectrum shows the presence of pyridinic-N (398.6 eV), quaternary-N (401.0 eV) and pyridine-N oxide (402.5 eV). The effective chemisorption of polysulfides has been demonstrated in pyridinic and pyrrolic groups where the N atoms have an electron-donating nature, as well as in the carbon atoms neighboring the N atoms with a quaternary configuration^34,35^. Furthermore, Yuan *et al*. showed that pyridinic-N not only possesses the strongest adsorption capacity for LiPSs, but it provides the lowest activation energy for decomposing Li_2_S to improve its utilization^12^. Meanwhile, the S 2p spectrum reveals that sulfur is present in the carbon framework as thiophenic sulfur (C-S-C, doublet at 164.1 and 165.3 eV) and oxidized sulfur (C-SO_x_-C, doublet at 168.0 and 169.2 eV). The presence of elemental sulfur (164.3 eV) is discarded since the carbon host was recovered by thermally treating the S/C composite at a high temperature of 600 °C. As with N, doping with S in the form of thiopenic sulfur has been shown to catalytically promote the conversion of LiPSs^13^.

**Table 1:** Physico-chemical properties of the porous carbon hosts and the corresponding sulfur/carbon composites.

Material	Sample code	S_BET_ (m^2^·g^−1^)	V_p_ (cm^3^·g^−1^)	V_micro <2 nm_ (cm^3^·g^−1^)^a^	S_infiltrated_ (wt%)^b^	S_doped_ (wt%)^c^	N_doped_ (wt%)^c^	Carbon yield (%)	Electrical conductivity (S·cm^−1^)^d^
S/carbon	CPS51	449	0.49	0.10	51				1.96
	CPS65	176	0.36	0.03	65				1.86
	CPS74	24	0.08	0.005	74				1.21
	CPS83	—	—	—	83				0.48
Porous carbon	CP51	2116	1.60	0.67		5.6	2.0	12.0	2.10
	CP65	2336	2.43	0.62		5.4	1.9	9.0	2.04
	CP74	2467	2.39	0.65		5.3	1.8	7.7	1.98
	CP83	2407	2.26	0.65		6.1	1.8	5.0	1.95

^a^The volume of micropores was calculated by using the QSDFT PSD; ^b^determined by thermogravimetric analysis; ^c^percentage of sulfur and nitrogen doped into the carbon framework, determined by elemental chemical analysis after removing the infiltrated sulfur; ^d^determined at 7.1 MPa.

**Figure 3:**
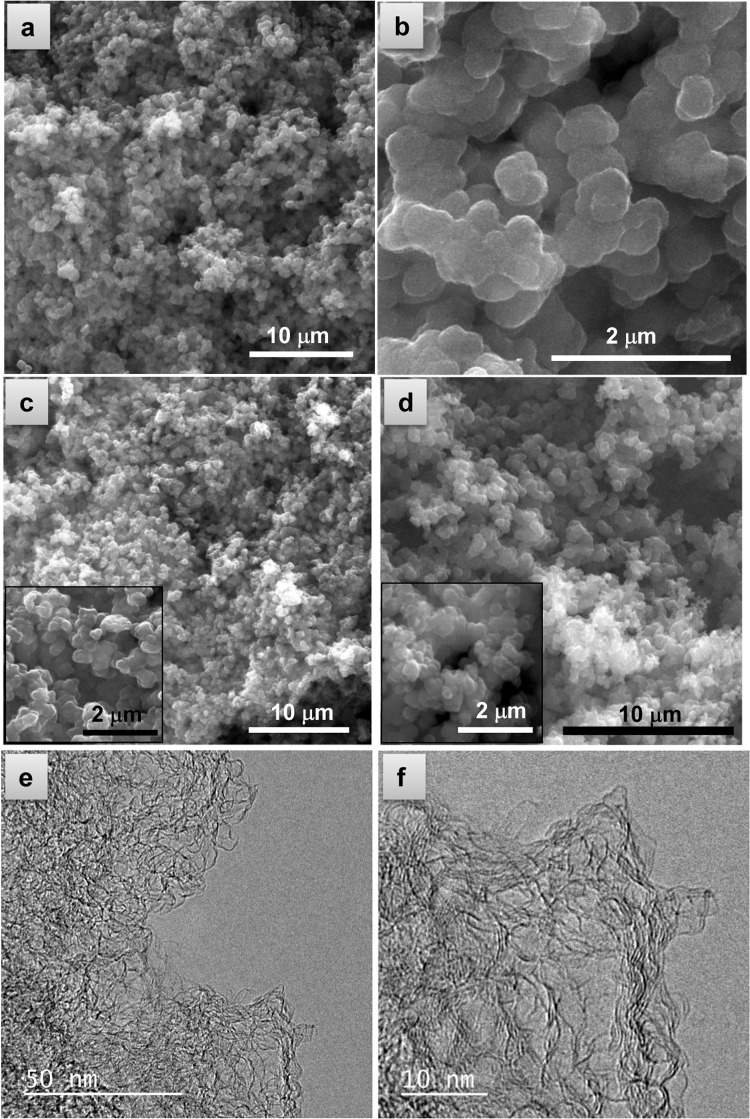
EM images of (**a**,**b**) CPS51, (**c**) CP51 and (**d**) polypyrrole precursor. (**e**,**f**) HRTEM images of CP51.

Further SEM inspection shows that both the composites and the carbon hosts exhibit a sponge-like structure formed by interconnected microparticles with a diameter of around 0.5–1 μm (Figs. 3a–c and S3). This morphology is similar to that of the polypyrrole used as precursor (Fig. 3d), which suggests that the activation strategy employed does not destroy the morphology of the precursor. The similarity between the surface of the particles in Figs. 3b and S3 (C/S composite) and that in the inset of Fig. 3c (porous carbon) further supports the effective infiltration of sulfur into the pore network of the carbon host. A closer examination of the porous carbon hosts by TEM reveals abundant mesopores of size <10 nm and separated by thin walls made up of a few graphene layers (Fig. 3e,f).

**Figure 4:**
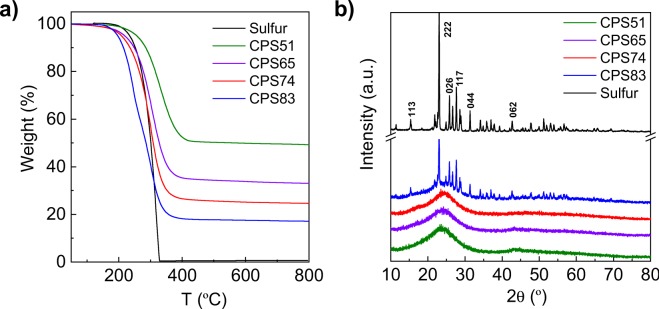
(**a**) TGA curves corresponding to the sulfur/carbon composites (N_2_, 5 °C/min) and (**b**) XRD patterns.

The sulfur content in the composites was determined by TGA and the corresponding curves are shown in Fig. 4a. An important characteristic of this synthesis approach is that it allows the precise modulation of the amount of sulfur introduced in the composite through the control of the amount of sodium thiosulfate used. In this way, the percentage of sulfur can be tailored from 24 wt% to *ca*. 90 wt% just by modifying the Na_2_S_2_O_3_/polypyrrole weight ratio between 1.5 and 3.2 (see Fig. S4). As the sulfur content rises, a reduction in the carbon yield takes places owing to the increased activation with the increase in the amount of thiosulfate. Figure 4a reveals a clear decrease in the temperature necessary for the sulfur to evaporate as the sulfur content in the composite increases, reflecting a weakening of the interactive forces between the sulfur and the carbon host. As will be shown later, this agrees with the infiltration of sulfur into mesopores besides micropores in the samples with higher amounts of sulfur. The XRD patterns of the sulfur/carbon composites are depicted in Fig. 4b. As can be seen, for sulfur contents lower than 74%, only a broad band in the range of 2θ from 15° to 30° is observed, which corresponds to the (002) band of amorphous carbon^36^. However, no diffraction peaks characteristic of crystalline sulfur are apparent, which lends further support to the probability that the sulfur is well dispersed and confined within the porous structure of the carbon material in the form of small nanoparticles. In contrast, for the composites with a sulfur content >80%, defined peaks assigned to orthorhombic sulfur (JCPDS 83-2283) are visible, which indicates that a fraction of sulfur is located outside the pores in the form of a crystalline phase. This is further confirmed by the Raman spectra shown in Fig. S5. The composites with a sulfur content <80% exhibit only two broad bands at ∼1350 cm^−1^ (D band) and ∼1600 cm^−1^ (G band), typical of carbon materials with a disordered structure. However, the spectrum of CPS83 (83% sulfur) contains three additional peaks at 154 cm^−1^, 219 cm^−1^ and 473 cm^−1^ associated with the S-S vibrational modes in crystalline sulfur.

Measurement of the electronic conductivity of the composites provides more hints as to the location of the sulfur in the composites. As can be seen in Table 1, for S contents below 74%, the composites have a good electronic conductivity of 1–2 S cm^−1^ (close to that of the carbon host), indicating that the insulating sulfur occupies the pore network and is not blocking the electron transport pathways between particles. However, for the highest sulfur content, *i.e*. 83%, a significant reduction in electronic conductivity is observed (0.48 S cm^−1^), suggesting that some sulfur is located on the surface of the particles.

**Figure 5:**
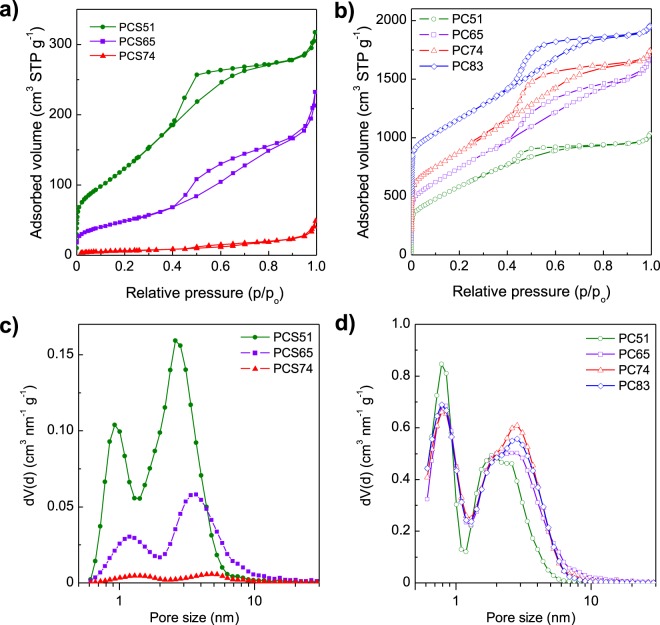
N_2_ sorption isotherms for (**a**) the S/C composites and (**b**) porous carbon hosts, and pore size distributions for (**c**) the S/C composites and (**d**) porous carbon hosts. The isotherms in (**b**) have been up-shifted for clarity.

The textural properties of the S/C composites and the corresponding porous carbon hosts were analyzed by N_2_ physisorption. Figure 5a,b show the nitrogen sorption isotherms for the composites and the carbon hosts respectively, whereas Fig. 5c,d show the pore size distributions (PSDs) and Table 1 the main textural properties. As can be seen from a comparison of their N_2_ isotherms, when the sulfur is loaded a drastic reduction in the adsorption capacity of the porous carbons takes place. This reduction occurs mainly in the region of the micropores, as can be deduced from the progressive disappearance of the sharp adsorption increase at P/P_o_ ~0 with the increase in sulfur loading in the composite. Meanwhile, the capillary condensation step associated with the presence of mesopores is maintained to a large extent. This is confirmed by comparing the PSDs of the composites and the corresponding carbon hosts (Fig. 5c,d). Thus, while in the porous carbons the population of pores of around 0.8 nm is greater than that of pores in the ~1.5–6 nm range, the opposite is the case in the S/C composites (in fact, there is an up-shift of the pore size maximums in the PSD of the composites). Taking into account the micropore volumes of the carbon hosts, *i.e*. 0.62–0.67 cm^3^ g^−1^ (see Table 1), a S content of 56–58% would be enough to completely fill their microporosity. This is confirmed by the micropore volumes of *ca*. 0 calculated for CPS65 and CPS74, whereas a micropore volume of 0.10 cm^3^ g^−1^ still remains in CPS51 which has a S content slightly lower than that of the theoretical value. Thus, all of the S is confined within the micropores in CPS51, whereas some of it is located in the mesopores in CPS65 (*i.e*., 9%) and CPS74 (*i.e*., 17%). In the case of CPS83, the S content is close to the maximum value that CP83 can host, *i.e*. 82%, so that all the pores are completely filled with S while some S is located on the surface of the carbon particles. This conclusion is in agreement with the results deduced from the XRD patterns, SEM/TEM inspection and TGA.

### Electrochemical performance of the sulfur/N-doped carbon composites as cathodes in Li-S batteries

To evaluate the electrochemical performance of the sulfur/N-S-doped carbon composites as cathodes in Li-S batteries, CR2032 coin cells were assembled using Li foil as the anode. The cyclic voltammograms of the composite with 51% S, *i.e*. CPS51, are shown in Fig. S6a. In the backward scan, during the first cycle two intense reduction peaks appear at 2.28 and 2.03 V (at 2.3 and 2.05 V in the successive cycles), and there is an additional small hump at 1.76 V. The intense reduction peak at the highest potential corresponds to the conversion of elemental sulfur to soluble long-chain lithium polysulfides (Li_2_S_X_, 4 ≤ x ≤ 8), while the peak at 2.03 V is attributed to the reduction of the LiPSs into solid Li_2_S_2_/Li_2_S^23^. On the other hand, the hump at 1.76 V, which disappears in successive cycles, can be ascribed to the irreversible reduction of the LiNO_3_ electrolyte additive^37^. Theoretically, assuming that all the LiPSs are reduced to Li_2_S, the area ratio of the peaks at 2.28 and 2.03 V should be 3. By integrating those peaks in the 2^nd^ cycle in Fig. S6a, a ratio of 2.4 is obtained, which indicates that most of the LiPSs have been successfully converted to Li_2_S_2_/Li_2_S. The forward scan also shows two intense peaks, which correspond to the oxidation of Li_2_S_2_/Li_2_S into soluble long-chain LiPSs (2.31–2.32 V) and their subsequent oxidation to sulfur (2.35–2.37 V). Integration of the areas of the forward and backward scans in the 2^nd^ cycle gives a high coulombic efficiency of ~95% (96–98% in the following cycles). Furthermore, the overlapping of the oxidation and reduction peaks during successive cycling indicates a good reversibility of the redox reactions. The carbon scaffold has a negligible contribution to capacity, as proved by cyclic voltammetry and galvanostatic charge-discharge experiments carried out for a battery built using the CP51 carbon as the cathode material (see Fig. S7).

**Figure 6:**
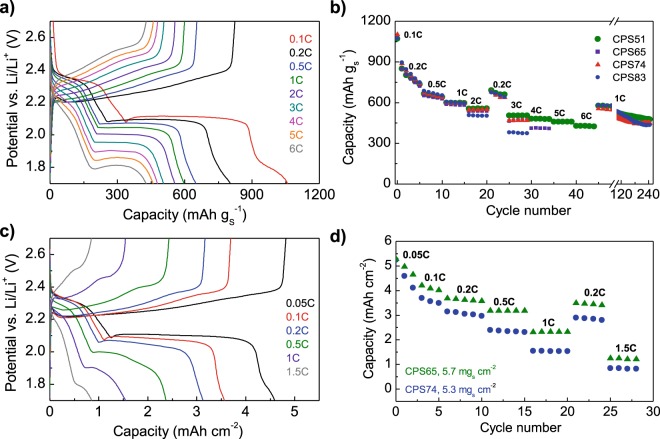
(**a**) Galvanostatic charge/discharge voltage profiles at different C-rates for CPS51 (areal S loading in the cathodes = 2 mg cm^−2^, electrolyte:S ratio = 30 μL mg^−1^), (**b**) C-rate performance of the sulfur/carbon composites with different sulfur contents (areal S loading in the cathodes = 2 mg cm^−2^, electrolyte:S ratio = 30 μL mg^−1^), (**c**) Galvanostatic charge/discharge voltage profiles at different C-rates for CPS74 (areal S loading in the cathode = 5.3 mg cm^−2^, electrolyte:S ratio = 20 μL mg^−1^) and (**d**) C-rate performance of high sulfur loading cathodes fabricated with CPS65 and CPS74 composites (electrolyte:S ratio = 20 μL mg^−1^).

Figure 6a shows the galvanostatic charge/discharge voltage profiles corresponding to CPS51 at an increasing C-rate from 0.1 C up to 6 C (1 C = 1675 mA h) in the potential range of 1.7–2.7 V. In agreement with the CV experiments, the discharge profiles show two typical plateaus consistent with the two-step reduction of sulfur described above. The increase in the C-rate is accompanied by increased polarization (*e.g*., from 180 mV at 0.2 C up to 490 mV at 3 C), which is a consequence of the sluggish reaction kinetics due to the insulating nature of the end-products S_8_ (~10^−30^ S cm^−1^) and Li_2_S (~10^−14^ S cm^−1^)^23^. This phenomenon is more marked with the increase in the S content in the composite (see Fig. S6b), as a result of reduced electron (lower electronic conductivity) and ion transport (reduced pore accessibility). Nevertheless, the redox processes are still discernable up to 6 C for the composite with 51% S (Fig. 6a) and up to 3 C for the composite with 83% S (Fig. S6c), which indicates fast reaction kinetics. This can be ascribed to the good electronic conductivity of the composites (Table 1) and to their favorable surface chemistry and micro-/mesoporous structure, which facilitate the redox accessibility of sulfur and the appropriate accommodation of the discharge products (Li_2_S_2_ and Li_2_S) at high current loads.

The rate capability of the cathodes with different sulfur contents is depicted in Fig. 6b. Regardless of the sulfur content –even up to 83%– the initial discharge capacity at 0.1 C is around 1100 mAh g^−1^ S, which corresponds to a sulfur utilization of *ca*. 70% based on the theoretical specific capacity of S (1675 mAh g^−1^). It is important to note that a reversible capacity ≥ 500 mAh g^−1^ S is still maintained at a high rate of 2 C independently of the S content in the composite, and a capacity of ~470–506 mAh g^−1^ S at 3 C for S contents ≤ 74%. Also worth noting is that a capacity as high as 430 mAh g^−1^ S is achieved at 6 C for the composite with 51% S (it should be noted that CPS65 cannot work at rates higher than 4C, and CPS74 and CPS83 at rates higher than 3C). In addition, a capacity of ~670 mAh g^−1^ S is recovered when all the cathodes are cycled back at 0.2 C, which suggests that the structure of the cathodes is not altered under high rates. Furthermore, further cycling at 1 C after the C-rate test confirms the stability of the developed cathodes, which are able to retain 75–82% of capacity (>98% coulombic efficiency) after an additional 200 cycles (the voltage profiles at different cycles are shown in Fig. S6d for CPS74). The rate performance of these materials compares well with other top-performing sulfur cathodes reported so far with similar sulfur loadings, and it is superior to that of many S/polypyrrole composites (see Table S1). Evaluation of the capacity per electrode instead of active material (*i.e*., sulfur), shows the importance of using composites with high sulfur content for achieving a high energy density battery (Fig. S8). Accordingly, to further evaluate the practical applicability of these cathodes, PCS65 and PCS74 were selected and used for the assembly of batteries with increased sulfur loading of above 5 mg S cm^−2^ and decreased electrolyte/S ratio of 20 μL mg^−1^. Figure 6c shows the corresponding galvanostatic charge/discharge cycles at different C-rates for CPSP74 (while Fig. S9a for CPS65). As can be seen, clear two-plateau voltage profiles are still achievable at high sulfur loadings up to 1.5 C, indicating good sulfur redox kinetics in these cathodes even under high sulfur loading conditions (the same behavior is observed for CPS65 in Fig. S9a). Even though the specific capacity decreases slightly (~10%) indicating that sulfur utilization has decreased (Fig. S9b), Fig. 6d shows that an initial areal capacity -which is the relevant parameter from the point of view of applicability- of 5.3 mA h cm^−2^ (930–1000 mA h g^−1^ S) is achieved for both cathodes at 0.05 C, and even 2.4 and 3.2 mA h cm^−2^ (value which is comparable to those of commercial Li-ion batteries, *i.e*. 3–4 mA h cm^−2^ ^38^) for CPS74 and CPS65 respectively with a 10-fold increase in current up to 0.5 C. As in the case of the low sulfur loading cathodes, when cycled back to 0.2 C, a reversible capacity of 2.8–3.5 mA h cm^−1^ is achieved, confirming the stability of the structure under high rates.

**Figure 7:**
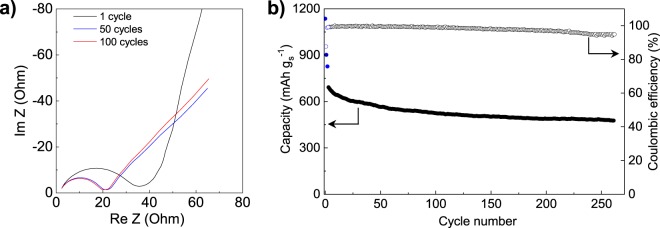
(**a**) Nyquist plots for CPS74, and (**b**) long-term cycling stability at 0.2 C for CPS74 (areal S loading in the cathode = 4 mg cm^−2^, electrolyte:S ratio = 20 μL mg^−1^).

The good electrochemical performance of these materials is supported by EIS analysis. Figure 7a shows the Nyquist plots for the composite CPS74 after 1, 50 and 100 cycles of galvanostatic charge/discharge at 1C. As can be seen, after 50 cycles, the charge-transfer resistance, which is reflected by the amplitude of the high frequency semicircle, decreases from *ca*. 33 to *ca*. 19 Ohm, indicating a good electrolyte infiltration and improved reaction kinetics. Upon further cycling, the resistance remains virtually constant, confirming the stable cyclability of the cell. Overall, the results suggest that the active material is efficiently redistributed during cycling, favoring both electron transfer at the carbon surface and ion transport through the electrode. The cycling stability of the batteries is further confirmed by long-term cycling at 0.2 C, even when using a cathode with a S content >70% (CPS74, 74% S) with an increased sulfur loading of 4 mg cm^−2^ and a reduced electrolyte/S ratio of 20 μL mg^−1^. Initial conditioning consisted of three cycles at 0.1 C (marked in blue). As can be seen in Fig. 7b, CPS74 is able to retain 70% of its capacity after 260 cycles (*i.e*., a loss of 0.12%/cycle), showing a rate loss of 0.23%/cycle during the first 100 cycles and of only 0.05%/cycle (0.006 mAh cm^−2^/cycle) during the following 160 cycles (final capacity of *ca*. 480 mAh g^−1^ S or 2 mAh cm^−2^). It should be also pointed out that the capacity ratio between the lower-discharge plateau (Q_L_) and the upper-discharge plateau (Q_H_) remains at around 2.1 throughout the duration of the cycling, which suggests an efficient retention of polysulfides in the cathode as well as a favorable Li^+^/e^−^ transport within the host (the theoretical value of Q_L_/Q_H_ is 3)^39,40^. This is further supported by a coulombic efficiency of above 97% throughout most of the stability study, which decreases only slightly to 94% towards the end.

## Conclusions

In summary, sulfur composite cathodes comprising high surface area, N,S-codoped carbons have been successfully synthesized by applying a simple, cost-effective and scalable synthesis procedure which makes use of a sulfur salt, *i.e*. sodium thiosulfate, as activating agent, sulfur precursor and S-dopant. This approach simplifies the fabrication process by combining the production of the porous host and the incorporation of sulfur in the same procedure. N-rich polypyrrole was selected as carbon precursor to allow additional N-doping of the sulfur host (~2 wt%). The porous hosts thus produced had BET surface areas in the ~2100–2500 m^2^ g^−1^ range and a micro-mesoporous structure. By simply controlling the amount of sodium thiosulfate used, it was possible to precise modulate the sulfur content in the composites in the range of 24 to *ca*. 90 wt%. The composites with sulfur contents in the 51–83 wt% range exhibited a good performance as cathodes in Li-S batteries, with high specific capacities of 1100 mAh g^−1^ S at a low rate and still ≥500 mAh g^−1^ S at a high rate of 2 C. The practical applicability of these cathodes was confirmed by assembling batteries with high sulfur content composites (65–74 wt%) and high sulfur loadings (5.3–5.7 mg cm^−2^). Even under these conditions the cathodes showed a good rate capability (300–400 mAh g^−1^ S at 1 C rate) and sulfur utilization (930–1000 mA h g^−1^ S/5.3 mA h cm^−2^ at 0.05 C). Their long-term stability was borne out by a capacity retention of 70% after 260 cycles at 0.2 C (i.e., a loss of 0.12%/cycle).

## Methods

### Synthesis of sulfur/N-doped carbon composites

In a first step, polypyrrole was produced from pyrrole by oxidative polymerization using FeCl_3_. In a typical synthesis, 7.5 g of pyrrole (Aldrich, recently distilled under N_2_) was added to a solution of FeCl_3_ (0.5 M, 500 mL) and the mixture was magnetically stirred for 2 h. The resultant polypyrrole was separated by filtration, washed with diluted HCl then washed again with abundant distilled water, and finally dried. The polypyrrole yield was around 100%. In a second step, the polypyrrole was physically mixed in a mortar with potassium chloride (Aldrich) and sodium thiosulfate (Alfa-Aesar). Afterwards, the mixture was heat-treated under N_2_ up to 800 °C at a heating rate of 5 °C min^−1^ and held at this temperature for 1 h. The solid product was then dispersed in 5 M HCl for 8 h inside a fume hood under continuous stirring, wherein the inorganic reaction products (*e.g*. KCl and Na_2_S) are dissolved and elemental sulfur and H_2_S is generated due to the disproportionation of the polysulfides generated during the activation. Finally, the sulfur/carbon composite was collected by filtration, washed with abundant distilled water and dried at 90 °C for three hours. In these experiments, different amounts of thiosulfate were used (the Na_2_S_2_O_3_/polypyrrole weight ratio ranged between 1.5 and 3.2), keeping the KCl/Na_2_S_2_O_3_ weight ratio at a value of 3. The resulting sulfur/carbon samples were denoted as CPSx, x being the sulfur fraction in the composite. The porous carbon particles were collected from the S/C samples by the removal of sulfur through heat treatment of the composites up to 600 °C under a N_2_ atmosphere. These porous carbon samples are designated as CPx.

### Physicochemical characterization

Scanning electron microscopy (SEM) images and energy dispersive X-ray analysis (EDX) were acquired by using a Quanta FEG650 (FEI) instrument, while high-resolution transmission electron microscopy (HRTEM) images were obtained on a JEOL (JEM 2100-F) apparatus operating at 200 kV. The electrical conductivity of the carbon powders was determined on a homemade apparatus in which the powders were placed between two plungers inside a hollow nylon cylinder (inner diameter of 8 mm), and then a pressure of 7.1 MPa was applied. The N_2_ sorption isotherms of the carbon samples were measured at −196 °C using a Micromeritics ASAP 2020 sorptometer. The apparent surface area was calculated by applying the BET method. An appropriate relative pressure range was selected to ensure a positive line intersect of multipoint BET fitting (C > 0). The total pore volume was determined from the amount of nitrogen adsorbed at a relative pressure (p/p_o_) of 0.99. The pore size distributions (PSD) were determined by means of the Quench Solid State Density Functional Theory (QSDFT) method for nitrogen. X-ray diffraction (XRD) patterns were obtained on a Siemens D5000 instrument operating at 40 kV and 20 mA, using a Cu-Kα radiation source. X-ray photoelectron spectroscopy (XPS) was carried out on a Specs spectrometer, using Mg KR (1253.6 eV) radiation from a double anode at 150 W. Calibration of the binding energies for the high resolution spectra was performed by setting the C 1 s signal to 284.5 eV. High resolution spectra were resolved into individual peaks and curve fitting was performed by an iterative least squares algorithm (CasaXPS software) using a Gaussian–Lorentzian (70/30) peak shape and applying the Shirley background correction. The S2p spectrum was deconvoluted on the basis of a separation between peaks in each of the doublets of ~1.2 eV and taking into account that the ratio of areas between the spin-up state, j3/2, and the spin-down state, j1/2, is equal to 2. The elemental analysis of the samples was carried out on a LECO CHN-932 microanalyzer. Thermogravimetric analysis (TGA) curves were recorded on a TA Instruments Q6000 TGA system.

### Electrochemical characterization

The sulfur/carbon composite (80 wt%), Super C65 carbon black (10 wt%) and PVDF binder (10 wt%) were mixed together and dispersed in N-methyl-2-pyrrolidone (NMP) by magnetic stirring to form thick slurries. The slurries were then cast onto Al foil using the Doctor Blade technique and dried at 50 °C under vacuum overnight. Afterwards, the coated foil was cut into discs 10 mm in diameter (areal S loading in the cathodes = 2 mg cm^−2^). CR2032 coin-type cells were assembled in an Ar filled glovebox using a Li metal foil as the anode and a Celgard 2500 membrane as the separator. The electrolyte consisted of a solution of 1 м lithium bis(tri-fluoromethane) sulfonimide (LiTFSI) in a mixed solvent of 1,3-dioxolane and 1,2-dimethoxyethane (DOL/DME, vol/vol = 1:1) with 1 wt.% LiNO_3_ (electrolyte:sulfur ratio = 30 μL mg^−1^). The electrochemical performance of the cells was tested at room temperature in a computer-controlled potentiostat (Biologic VMP3 multichannel generator). Galvanostatic charge/discharge (CD) experiments were recorded over a potential range of 1.7–2.7 V (*vs*. Li^+^/Li) at different C rates in the 0.1 to 6 C range (1 C = 1675 mA g^−1^ S). Cyclic voltammetry (CV) experiments were recorded within the same potential window at a sweep rate of 0.05 mV s^−1^. Electrochemical impedance spectroscopy (EIS) curves were obtained at open-circuit potential after the first discharge within a frequency range of 100 kHz-1 mHz applying an amplitude of 10 mV.

## Supplementary information

Supplementary Information.
