# Supplementary material for: Straightforward synthesis of Sulfur/N,S-codoped carbon cathodes for Lithium-Sulfur batteries
Source: Sci Rep. 2020 Mar 17;10:4866. doi: 10.1038/s41598-020-61583-1 (PMC7078249; doi:10.1038/s41598-020-61583-1)
Supplement: Supplementary file 1 — Supplementary Information. [file 41598_2020_61583_MOESM1_ESM.docx]

**Straightforward synthesis of Sulfur/N,S-codoped carbon cathodes for Lithium-Sulfur batteries**

**Marta Sevilla,^*^ Jorge Carro-Rodríguez, Noel Díez,^*^ Antonio B. Fuertes**

*Instituto Nacional del Carbón (CSIC), Fco. Pintado Fe 26, Oviedo 33011, Spain*

*** martasev@incar.csic.es, noel@incar.csic.es**

**Figure S1.** SEM-EDX mappings of a) nitrogen, b) carbon, c) sulfur and d) oxygen collected from the area shown in (a) for the sample CP51.

**Figure S2.** XPS high resolution (a) N 1s and (b) S 2p core level spectra obtained for the carbon material.

**Figure S3.** SEM images of CPS74.

**Figure S4.** Variation of the sulfur content in the composite and carbon yield with the increase in the Na_2_S_2_O_3_/polypyrrole weight ratio.

**Figure S5.** Raman spectra of the PCS samples. The spectrum of commercial sulfur powder is also included as a reference.

**Figure S6.** a) Cyclic voltammograms of CPS51, b) comparison of the galvanostatic charge/discharge voltage profiles at a low and a high rate for composites with different sulfur loadings (51 and 74 %), c) galvanostatic charge/discharge voltage profiles at an increasing rate for CPS83 and d) in different cycles during long-term cycling at 1C for CPS74.

**Figure S7.** Electrochemical characterization of the Li-S battery using the carbon CP51 as the cathode material: (a) Cyclic voltammetry at 0.05 mV s^-1^ (10^th^ cycle; the plot corresponding to the CPS51 composite is included as a reference); (b) galvanostatic charge-discharge profiles and (c) capacity retention during 500 cycles of charge-discharge.

**0.1C**

**0.2C**

**0.5C**

**1C**

**2C**

**0.2C**

**3C**

**4C**

**1C**

**5C**

**6C**

**Figure S8.** C-rate performance of the sulfur/carbon composites with different sulfur contents (areal S loading in the cathodes = 2 mg cm^−2^, electrolyte: S ratio = 30 μL mg^-1^), evaluated per electrode.

**Figure S9.** a) Galvanostatic charge/discharge voltage profiles for CPS65 under high sulfur loading conditions (5.7 mg_s_ cm^-2^) and b) evolution of the specific capacity with an increase in current for CPS65 and CPS74 under high sulfur loading conditions (electrolyte/S ratio = 20 μL mg^-1^).

**Table S1.** Electrochemical performance of Li-S cells reported in the literature made from different sulfur composites but similar sulfur loadings.

| **Sample** | **Sulfur content**  **(wt. %)** | **Sulfur loading**  **(mg cm^-2^)** | **Capacity (mA h g_S_^-1^)** | | | | | **Reference** |
| --- | --- | --- | --- | --- | --- | --- | --- | --- |
|  |  |  | **0.1C** | **0.2C** | **1C** | **2C** | **3C** |  |
| S-PPy | 77 | 1-2^a^ | - | - | 500 | 200 | - | ^1^ |
| AB/S/PPy | 59 | 1.5 | - | 1059 | 408 | - | - | ^2^ |
| PPy@S@PPy | 65.6 | n.s. | 600 | 486 | 295 | - | - | ^3^ |
| PPy/S@PPy | < 53 | 1.4 | 1200 | 908 | 650 | - | - | ^4^ |
| PPy@S/Fe-3 | 61.3 | n.s. | 850 | 778 | 445 | 322 | - | ^5^ |
| PPy@S@GA | 56.6 | 1.0-2.0 | 1218 | 1069 | 741 | 643 |  | ^6^ |
| S@GA | 79 |  | 1098 | 891 | 694 | 600 |  |  |
| S/MMCS | 60.6 | 1.0 | 1500 | 1108 | 876 | 648 | - | ^7^ |
| PGF-S | 63.2 | 2.4 | 1265 | 1056 | 806 | 748 | - | ^8^ |
| HPC-S-85 | 85 | 1.0-1.2 | - | ~1030 | ~670 | 619 | 580 | ^9^ |
| S@PGC | 90 | *ca*. 2.36 | - | 1427 | 1154 | 1038 | 890 | ^10^ |
| NPMCG/S-80/G | 80 | 2.6 | 1296 | 1050 | 768 | 592 | 445 | ^11^ |
| Act PyLs-Cap S+S | 68 | 2.0 | - | 1109 | 720 | 647 | 468 | ^12^ |
| CPS-83 | 83 | 2.0 | 1074 | 896 | 605 | 507 | 380 | This work |
| CPS-74 | 74 |  | 1100 | 890 | 602 | 550 | 470 |  |

^a^ Sulfur loading is provided in mg.

1 Fu, Y., Su, Y.S. & Manthiram, A. Sulfur-polypyrrole composite cathodes for lithium-sulfur batteries. *J. Electrochem. Soc.* **159**, A1420-A1424 (2012).

2 Li, S. *et al.* Improved electrochemical performance of Li-S battery with carbon and polymer-modified cathode. *Appl. Surf. Sci.* **479**, 265-272, 10.1016/j.apsusc.2019.02.109 (2019).

3 Liang, X. *et al.* Split-half-tubular polypyrrole@sulfur@polypyrrole composite with a novel three-layer-3D structure as cathode for lithium/sulfur batteries. *Nano Energy* **11**, 587-599, 10.1016/j.nanoen.2014.10.009 (2015).

4 Li, F. *et al.* Free-standing sulfur-polypyrrole cathode in conjunction with polypyrrole-coated separator for flexible Li-S batteries. *Energy Storage Mater.* **13**, 312-322 (2018).

5 Wei, W., Du, P., Liu, D., Wang, Q. & Liu, P. Facile one-pot synthesis of well-defined coaxial sulfur/polypyrrole tubular nanocomposites as cathodes for long-cycling lithium–sulfur batteries. *Nanoscale* **10**, 13037-13044, 10.1039/C8NR01530A (2018).

6 Tang, H. *et al.* Integrated polypyrrole@sulfur@graphene aerogel 3D architecture via advanced vapor polymerization for high-performance lithium–sulfur batteries. *ACS App. Mater. Inter.* **11**, 18448-18455, 10.1021/acsami.9b04167 (2019).

7 Li, Z. *et al.* A highly ordered meso@microporous carbon-supported sulfur@smaller sulfur core–shell structured cathode for Li–S batteries. *ACS Nano* **8**, 9295-9303, 10.1021/nn503220h (2014).

8 Shi, J.L. *et al.* 3D mesoporous graphene: CVD self-assembly on porous oxide templates and applications in high-stable Li-S batteries. *Small* **11**, 5243-5252, 10.1002/smll.201501467 (2015).

9 Luo, C. *et al.* A dual-function Na_2_SO_4_ template directed formation of cathode materials with a high content of sulfur nanodots for lithium–sulfur batteries. *Small* **13**, 1700358, 10.1002/smll.201700358 (2017).

10 Li, G. *et al.* Three-dimensional porous carbon composites containing high sulfur nanoparticle content for high-performance lithium–sulfur batteries. *Nat. Commun.* **7**, 10601, 10.1038/ncomms10601 (2016).

11 Zhao, Q. *et al.* High sulfur loading, rGO-linked and polymer binder-free cathodes based on rGO wrapped N,P-codoped mesoporous carbon as sulfur host for Li-S batteries. *Chem. Eng. J.* **361**, 1043-1052, 10.1016/j.cej.2018.12.153 (2019).

12 Li, L., Huang, L., Linhardt, R. J., Koratkar, N. & Simmons, T. Repurposing paper by-product lignosulfonate as a sulfur donor/acceptor for high performance lithium–sulfur batteries. *Sustain. Energy Fuels* **2**, 422-429, 10.1039/C7SE00394C (2018).
